# Unraveling metabolic characteristics and clinical implications in gastric cancer through single-cell resolution analysis

**DOI:** 10.3389/fmolb.2024.1399679

**Published:** 2024-05-20

**Authors:** Wenyue Wang, Conghui Li, Yuting Dai, Qingfa Wu, Weiqiang Yu

**Affiliations:** ^1^ School of Life Sciences, Tianjin University, Tianjin, China; ^2^ HIM-BGI Omics Center, Zhejiang Cancer Hospital, Hangzhou Institute of Medicine (HIM), Chinese Academy of Sciences (CAS), Hangzhou, China; ^3^ Department of Biology, University of Copenhagen, Copenhagen, Denmark; ^4^ Center for Advanced Interdisciplinary Science and Biomedicine of IHM, Division of Life Sciences and Medicine, University of Science and Technology of China, Hefei, Anhui, China; ^5^ Department of Pharmacy, The First Affiliated Hospital of USTC, Division of Life Sciences and Medicine, University of Science and Technology of China, Hefei, Anhui, China

**Keywords:** gastric cancer, scRNA-seq, malignant cell, metabolic pathway, prognosis

## Abstract

**Background:** Gastric cancer is a highly prevalent malignant neoplasm. Metabolic reprogramming is intricately linked to both tumorigenesis and cancer immune evasion. The advent of single-cell RNA sequencing technology provides a novel perspective for evaluating cellular metabolism. This study aims to comprehensively investigate the metabolic pathways of various cell types in tumor and normal samples at high resolution and delve into the intricate regulatory mechanisms governing the metabolic activity of malignant cells in gastric cancer.

**Methods:** Utilizing single-cell RNA sequencing data from gastric cancer, we constructed metabolic landscape maps for different cell types in tumor and normal samples. Employing unsupervised clustering, we categorized malignant cells in tumor samples into high and low metabolic subclusters and further explored the characteristics of these subclusters.

**Results:** Our research findings indicate that epithelial cells in tumor samples exhibit significantly higher activity in most KEGG metabolic pathways compared to other cell types. Unsupervised clustering, based on the scores of metabolic pathways, classified malignant cells into high and low metabolic subclusters. In the high metabolic subcluster, it demonstrated the potential to induce a stronger immune response, correlating with a relatively favorable prognosis. In the low metabolic subcluster, a subset of cells resembling cancer stem cells (CSCs) was identified, and its prognosis was less favorable. Furthermore, a set of risk genes associated with this subcluster was discovered.

**Conclusion:** This study reveals the intricate regulatory mechanisms governing the metabolic activity of malignant cells in gastric cancer, offering new perspectives for improving prognosis and treatment strategies.

## Introduction

Gastric cancer is one of the most common malignancies globally, ranking fifth in incidence and fourth in mortality rates worldwide. In 2020, there were over one million new cases of gastric cancer, with an estimated 769,000 deaths (Tang et al., 2021). Adenocarcinoma is the predominant histological subtype of gastric cancer, accounting for approximately 95% of cases (Hwang et al., 2010). Other classifications include the Lauren classification (Lauren, 1965) (intestinal type, diffuse type, mixed type) and the WHO classification (Bosman et al., 2010) (papillary, tubular, mucinous and poorly cohesive carcinomas). The Cancer Genome Atlas (TCGA) project uncovered four molecular subtypes of gastric cancer: Epstein-Barr virus (EBV), microsatellite instability (MSI), genomically stable (GS), and chromosomal instability (CIN) (Comprehensive molecular characterization of gastric, 2014). This indicates that gastric cancer is a highly heterogeneous tumor.

Metabolic reprogramming is one of the hallmarks of cancer and is intricately linked to both tumorigenesis and cancer immune evasion (Faubert et al., 2020; Gong et al., 2021). Throughout the progression of a tumor, cancer cells encounter diverse metabolic challenges. For instance, Cancer cells must compete for nutrients with various other cell types in the tumor microenvironment (TME) (Martínez-Reyes and Chandel, 2021; Ohshima and Morii, 2021). Research has reported that the increased levels of the transporter protein (SLC43A2) in cancer cells result in elevated consumption of methionine, restricting methionine metabolism in cytotoxic T cells (CTLs), which impairs their function (Sullivan et al., 2019; Bian et al., 2020). Additionally, malignant cells undergoing metastasis need to survive in the circulatory or lymphatic system in order to reach and colonize distal sites. During this process, cancer cells are not in an anabolic state but, rather, enter a catabolic state in order to survive the changing environment (Ubellacker and Morrison, 2019).

Numerous prior studies have provided substantial evidence establishing the link between metabolic dysregulation and clinical outcomes, as well as treatment responses, across various cancer types (Liu et al., 2018; Gong et al., 2021). Comprehensively understanding cancer metabolism necessitates insights into both metabolite concentrations and transformation rates, although acquiring these measurements in humans presents challenges. While the expression levels of metabolic genes may not directly correlate with metabolic flux or metabolite abundance, evidence suggests that metabolic gene expression can offer valuable insights into predicting metabolic flux and metabolite concentrations (Mehrmohamadi et al., 2014). Compared with the bulk RNA-seq, single-cell RNA sequencing (scRNA-seq) could provide some insights into metabolism at the single-cell level in human tumors (Xiao et al., 2019). This presents a unique opportunity to gain in-depth insights into the metabolic activities of various cell subgroups within cancer tissues.

Currently, scRNA-seq in cancer research has predominantly concentrated on the identification and comparison of functional cell subpopulations. Limited studies have explored tumor metabolic heterogeneity using single-cell RNA sequencing data. In a recent study, researchers systematically delineated the intricate single-cell landscape of gastric cancer, addressing both inter- and intratumoral heterogeneity (Kumar et al., 2022). Leveraging the public available high quality scRNA-seq data, we conducted an in-depth exploration of the metabolic characteristics of various cell subpopulations in gastric cancers. Subsequently, we classified tumor cells into high-metabolism and low-metabolism subclusters based on their metabolic pathway scores. The high metabolic subcluster demonstrated the potential to induce a robust immune response, correlating with a relatively favorable prognosis. Conversely, within the low metabolic subcluster, a subset of cells exhibiting cancer stem cells (CSCs)-like characteristics correlated with a relatively poor prognosis. Our study unveils novel insights into the metabolic characteristics of both malignant and non-malignant cells in gastric cancer, providing valuable clues for predicting patient prognosis.

## Materials and methods

### Processing of scRNA-seq data

This study single-cell transcriptomic data for gastric cancer from the Gene Expression Omnibus (GEO; https://www.ncbi.nlm.nih.gov/gds) under the accession number GSE183904. The Unique Molecular Identifiers (UMIs) count matrix was processed using the R package Seurat (version 4.3.0). The criteria for cell filtering are as follows: a minimum gene count threshold of 500, a minimum threshold of 1,000 for UMIs, and a threshold of 20% for the percentage of mitochondrial genes out of the total gene count. Cells that do not meet these criteria are considered low-quality cells and are removed. Cell doublets were removed using the DoubletFinder package (version 2.0.3). Then, we used the merge function in R (version 4.2.1) to combine all quality-controlled sample expression profiles. To eliminate batch effects in single-cell RNA sequencing data, we employed the harmony package (version 0.1.1). After that, highly variable genes (HVGs) were selected for principal components analysis (PCA), and the top 30 significant principal components (PCs) were selected for Uniform Manifold Approximation and Projection (UMAP) and visualization of gene expression.

### Determination of cell type

The differentially expressed genes (DEGs) in each cell subcluster were identified using the “FindAllMarker” function provided by Seurat with the parameters min. pct = 0.25 and logfc. threshold = 0.25. Cell types were annotated based on the expression of canonical marker genes known for those cell types. Cell subclusters with similar gene expression patterns were annotated as the same cell type.

### Construction of the metabolic landscape atlas

The construction of the metabolic landscape atlas is primarily based on the metabolic scoring method proposed by Xiao and colleagues (Xiao et al., 2019). First, data imputation was conducted to address the drawbacks of “dropout” in single-cell sequencing. To obtain the true expression of these genes in cells as much as possible, the scImpute algorithm was used to impute the missing gene expression values (Li and Li, 2018), with Transcripts per million (TPM) values and gene lengths as inputs. Imputation was only applied to genes with dropout rates exceeding 50%.

Specific parameters are as follows: count_path, the full path of the raw count matrix; infile = “csv”: specifies the file type storing the raw count matrix as CSV; outfile = “csv”: specifies the file type storing the imputed count matrix as CSV; out_dir: specifies the folder path to store the imputed count matrix; labeled = TRUE: indicates whether cell type information is provided; labels: the result of single-cell clustering, representing cell types; type = “TPM”: specifies the numerical type in the expression matrix as TPM; genelen: the length information of each gene in the single-cell dataset; drop_thre = 0.5, sets the dropout threshold to 0.5, meaning that the scImpute algorithm will be used for imputation only when a gene is not expressed in more than half of the cells; ncores = 20, specifies the number of cores used for parallel computation as 20.

Metabolic gene and pathway information was obtained from the KEGG database. Some improvements were made in data imputation in this study. In previous research, imputation for non-malignant cells was based on cell-type similarity, and for malignant cells, it was based on patient heterogeneity, possibly due to limitations in the number of cells. To comprehensively consider various factors including patient heterogeneity, disease types, and disease progression, we believe that sample-level data imputation is a more reasonable approach when dealing with sufficiently rich single-cell data.

Following the completion of the imputation process, to ensure comparability between different samples or experimental conditions and accurately reflect biological differences, we standardized the data. Four methods were evaluated, including the relative log expression (RLE) method implemented in the estimate size factors for matrix function of the DESeq2 package, the TMM and upper quartile methods executed by the calcNormFactors function of the edgeR package, and the deconvolution method implemented in the computeSumFactors function of the scran package, with the latter demonstrating the best performance.

Calculation of metabolic pathway activity involved computing the average expression level of each gene in each cell type and comparing it with the average across all cell types to obtain relative expression levels. Pathway activity scores for each pathway in each cell type were defined using weighted averages, where the weighting factor is the reciprocal of the number of pathways containing that gene. Outliers were excluded, and pathway activity significance in specific cell types was assessed through random permutation tests.

### Calculation of metabolic pathway enrichment score

Metabolic pathway data were obtained from the Kyoto Encyclopedia of Genes and Genomes (KEGG) database (Kanehisa and Goto, 2000) (https://www.genome.jp/kegg/). A total of 1,667 human metabolic genes are distributed across 85 metabolic pathways (Supplementary Table S1). These pathways were categorized into 11 major classes based on KEGG classifications. We employed the Gene Set Enrichment Analysis (GSEA) (version 1.24.0) method (Subramanian et al., 2005) to score the pathways in malignant cells of tumor samples relative to normal samples, specifically focusing on epithelial cells. The differentially expressed genes were identified using presto (version 1.0.0), which utilizes the wilcoxauc function to calculate the ranked AUC for GSEA input. Additionally, we utilized Gene Set Variation Analysis (GSVA) (version 1.46.0) (Hänzelmann et al., 2013) to calculate the enrichment scores of each metabolic pathway in individual cells with transcriptomic data.

### Clustering cells based on metabolic scoring

First, the Euclidean distance matrix between cells is calculated using the dist function. Then, hierarchical clustering is performed on the distance matrix using the hclust function. Default parameters are used, and the complete linkage method is employed. Finally, the hierarchical clustering result is divided into two clusters using the cutree function. The functions of dist, hclust, and cutree are built-in functions in R (version 4.2.1).

### Re-clustering of the low metabolic cluster

First, we extracted cells from the original Seurat object that belong to the low metabolic cluster and constructed a new Seurat object. Next, we applied the SCT method for data standardization and normalization. Then, we used the Harmony package (version 0.1.1) to perform batch effect correction based on patient information. Highly variable genes (HVGs) were selected for principal component analysis (PCA), and the top 10 significant PCs were chosen for UMAP visualization. During the re-clustering of the low metabolic cluster, a resolution of 0.1 was selected. Finally, we used the “FindAllMarker” function provided by Seurat, setting the parameters min. pct = 0.25 and logfc. threshold = 0.25, to identify the DEGs within each cell cluster.

### Single-cell gene set enrichment analysis

Gene set enrichment analysis was conducted based on a modified version of the competitive gene set enrichment test CAMERA developed by Cillo et al. (Cillo et al., 2020), which has been incorporated into the SingleSeqGset R package (version 0.1.2). The SingleSeqGset package is designed for gene set enrichment analysis in single-cell RNA-seq data. It utilizes straightforward basic statistics, such as variance-inflated Wilcoxon rank-sum test, to determine the enrichment of gene sets of interest in different clusters. In this method, the mean gene expression level was initially computed, and the log2 fold change (FC) between the specific cell cluster and the other cells was employed as the test statistic (Cillo et al., 2020). Gene set enrichment analysis (GSEA) was performed using 50 hallmark gene sets from the MSigDB databases (https://www.gsea-msigdb.org/gsea/msigdb).

### Pseudotime analysis

In the low metabolic subcluster, 500 cells were randomly selected from six subclusters for pseudotime analysis using the Monocle two package (version 2.28.0) (Trapnell et al., 2014). The trajectory is visualized in the form of a 2D *t*-Distributed Stochastic Neighbor Embedding (tSNE) plot. A pivotal feature of monocle2, the plot_genes_in_pseudotime function, facilitated the visualization of gene expression changes along pseudotime, representing the inferred developmental trajectory of individual cells.

### Survival analysis

First, the code selects a specific gene set and computes expression scores for each sample in the TCGA dataset using GSVA. Then, we utilize the surv_cutpoint function from the survminer package (version 0.4.9) to determine the optimal grouping strategy for patients. Finally, survival analysis is conducted using the survival package (version 3.5.3) and survminer package to explore the correlation between the expression of these genes in patients and their prognosis. Bulk RNA-seq data for gastric cancer (FPKM), along with patient clinical data, was sourced from the UCSC Xena database website (https://xenabrowser.net/datapages/), comprising a total of 375 tumor samples.

## Results

### Cell types identified in both gastric cancer tumor and normal samples

**FIGURE 1:**
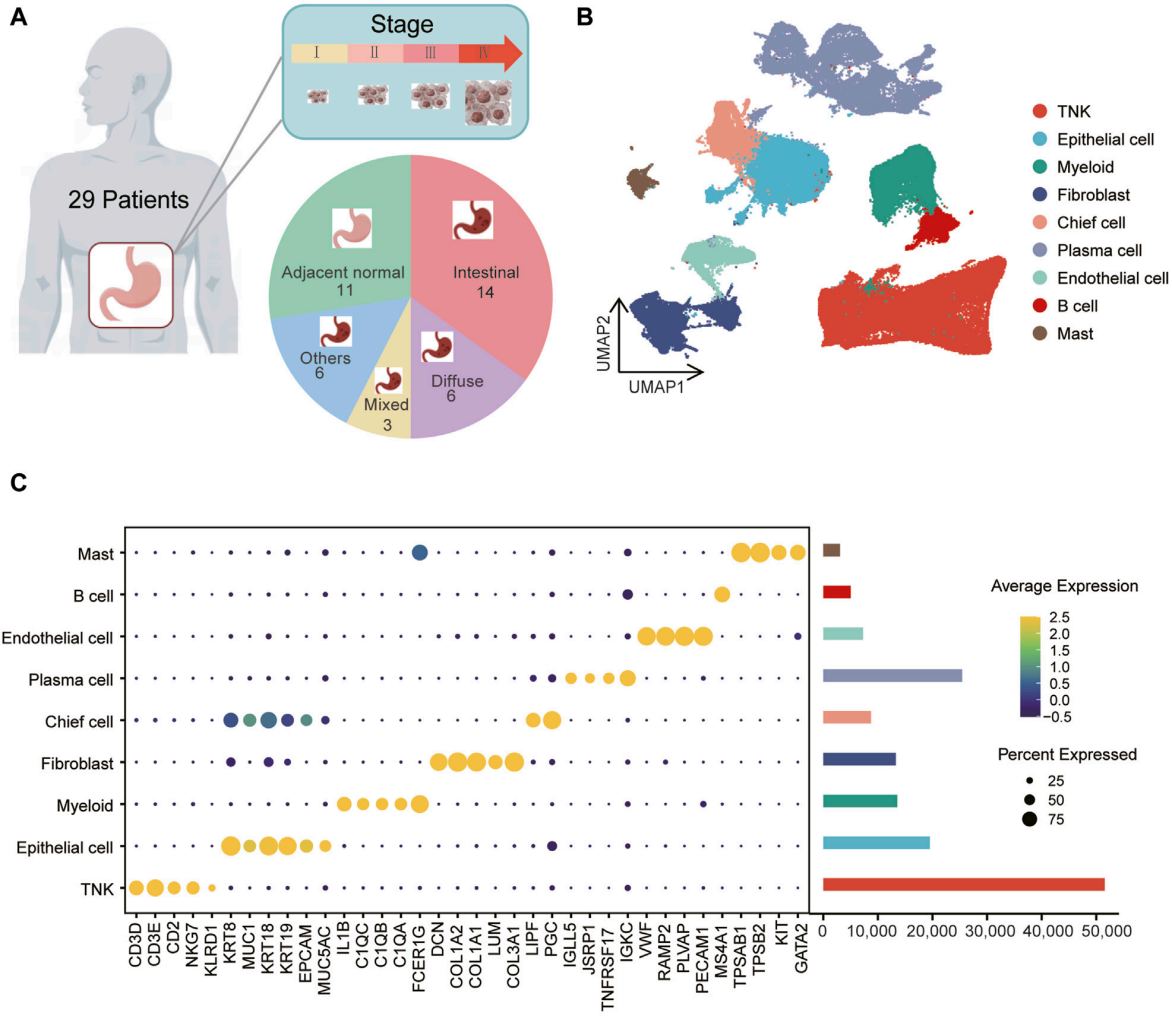
Identification of gastric cancer cell populations at single-cell resolution **(A)** The data includes information on 40 samples, consisting of 29 tumor samples and 11 normal samples, spanning across one to four clinical stages and various tissue types. **(B)** Uniform Manifold Approximation and Projection (UMAP) of 147,495 cells representing nine unique cell clusters, color-coded by their corresponding cell lineage or subtype. Each dot in the UMAP represents a single cell. **(C)** The left bubble plot illustrates marker genes specific to each cell cluster, while the right bar plot displays cell counts within each cell type.

In a prior cohort of 40 samples, including 29 tumor and 11 normal tissue samples (Figure 1A, Supplementary Table S2), scRNA-seq was employed to investigate single-cell landscape of gastric cancer (Kumar et al., 2022). Utilizing the scRNA-seq data (GEO accession: GSE183904), we investigated the metabolic characteristics of various cell subpopulations in gastric cancers. After quality control, we obtained 147,495 cells, including 118,009 from tumor tissues and 29,486 from normal tissues. Using dimensionality reduction and unsupervised clustering, we identified nine distinct cell types (Figure 1B). The predominant group was immune cells (Figure 1C), with TNK cells expressing *CD3D*, *CD3E, CD2, NKG7, KLRD1*, myeloid cells expressing *IL1B, C1QC, C1QB, C1QA, FCER1G*, plasma cells expressing *IGLL5, JSRP1, TNFRSF17, IGKC*, mast cells expressing *TPSAB1, TPSB2, KIT, GATA2*, and B cells expressing *MS4A1*. Stromal cells, primarily fibroblasts expressing *DCN, COL1A2, COL1A1, LUM, COL3A1*, and endothelial cells expressing *VWF, RAMP2, PECAM1, PLVAP*, were also identified (Figure 1C; Supplementary Figure S1A). Additionally, epithelial cells (labeled as *“EPCAM,” “KRT18,” “KRT19,” “MUC1,” “MUC5AC,” “KRT8”*) exhibited heterogeneity (Supplementary Figure S2A), forming two subclusters, cluster one and cluster 5, with cluster five showing higher expression of chief cell markers *LIPF* and *PGC* (Supplementary Figure S2B, C), leading to its annotation as chief cells. Notably, none of the cell types exhibited significant differences in proportions between gastric cancer tumor and normal samples (Supplementary Figure S3A, B).

### The metabolic landscape of cell types identified in gastric cancer

**FIGURE 2:**
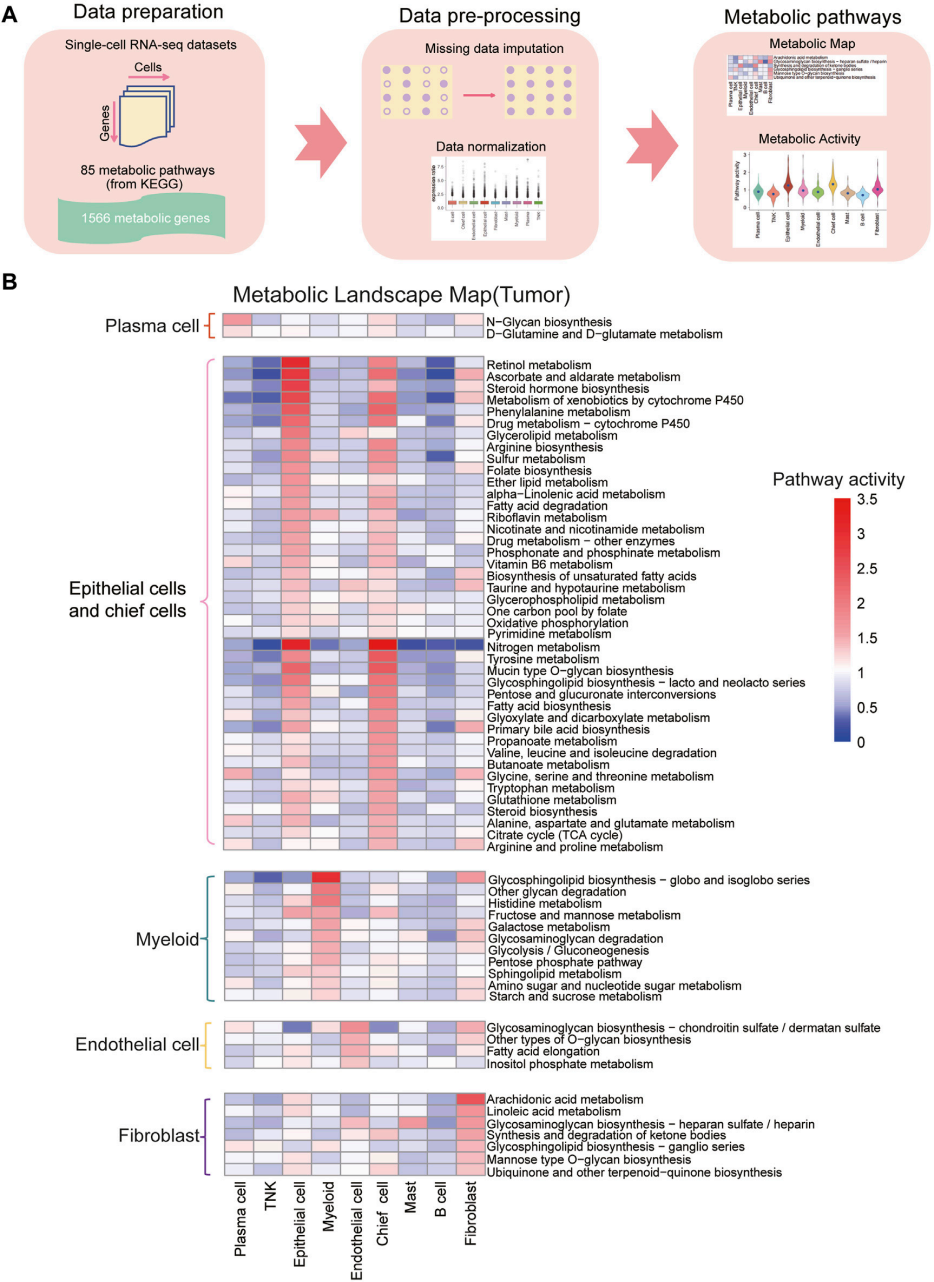
Construction of metabolic landscape at single-cell resolution **(A)** Schematic representation of the analysis pipeline for constructing the metabolic landscape at single-cell resolution. **(B)** Metabolic pathway activities across cell types in gastric cancer. Statistically non-significant values (random permutation test *padj* > 0.05) are depicted as blank. Pathways specific to plasma cells are highlighted in red boxes, those specific to epithelial cells and fibroblasts in pink boxes, myeloid-specific pathways in green boxes, endothelial cell-specific pathways in yellow boxes, and fibroblast-specific pathways in purple boxes.

To assess metabolic pathway characteristics across cell types in gastric cancer tumor and normal samples, we utilized 85 KEGG metabolic pathways. To address the issue of “dropout” in scRNA-seq, we applied the scImpute algorithm to impute the expression levels of dropout genes based on the same cell types within each sample (Li and Li, 2018). Subsequently, we scored the relative metabolic activity of each KEGG metabolic pathway in each cell type, and the analysis pipeline is illustrated in Figure 2A. The results revealed that, in both normal and tumor samples, epithelial cells and chief cells exhibited significantly higher metabolic activities compared to other cell types (Figure 2B and Supplementary Figure S4A, B; Supplementary Tables S3–S6, *padj* < 0.05). Notably, the retinol metabolism pathway, ascorbate and aldarate metabolism pathway, nitrogen metabolism, and fatty acid metabolic pathway displayed increased activity in the epithelial cells of tumor samples. This was accompanied by enhanced amino acid metabolism, distinguishing them from other cell types within the tumor samples (Supplementary Tables S3–S6, *padj* < 0.05).

Our study also revealed notable metabolic pathway activity in myeloid cells in both normal and tumor samples. Particularly, the glycosphingolipid biosynthesis pathway demonstrated significantly higher activity in myeloid cells compared to other cell types, suggesting their specific glycolipid synthesis capabilities for immune regulation and inflammatory processes (Leone and Powell, 2020). Additional pathways, including histidine metabolism, fructose and mannose metabolism, galactose metabolism, glycosaminoglycan degradation, glycolysis/gluconeogenesis, pentose phosphate pathway, sphingolipid metabolism, amino sugar and nucleotide sugar metabolism, and starch and sucrose metabolism, also exhibited elevated activity in myeloid cells compared to other cell types. In plasma cells, N-glycan biosynthesis and D-glutamine and D-glutamate metabolism displayed the highest metabolic activity compared to other cell types. Furthermore, endothelial cells demonstrated increased activity in glycosaminoglycan biosynthesis-chondroitin sulfate/dermatan sulfate, other types of O-glycan biosynthesis, fatty acid elongation, and inositol phosphate metabolism compared to other cell types.

Fibroblast cells exhibited elevated activity in arachidonic acid metabolism, linoleic acid metabolism, glycosaminoglycan biosynthesis-heparan sulfate/heparin, synthesis and degradation of ketone bodies, glycosphingolipid biosynthesis-ganglio series, mannose type O-glycan biosynthesis, ubiquinone and other terpenoid-quinone biosynthesis, particularly arachidonic acid metabolism, compared to other cell types. In tumor samples, fibroblast cells play a crucial role in the tumor microenvironment, contributing to processes such as tumor-related fibrosis, extracellular matrix remodeling, and angiogenesis (Kalluri, 2016; Hanna and Hafez, 2018). The increased metabolism of arachidonic acid could be linked to the specific functions of fibroblast cells in regulating the tumor microenvironment, supporting tumor growth and invasion, and participating in inflammatory responses (Hanna and Hafez, 2018).

### Dysregulation of diverse metabolism pathways in malignant epithelial cells

**FIGURE 3:**
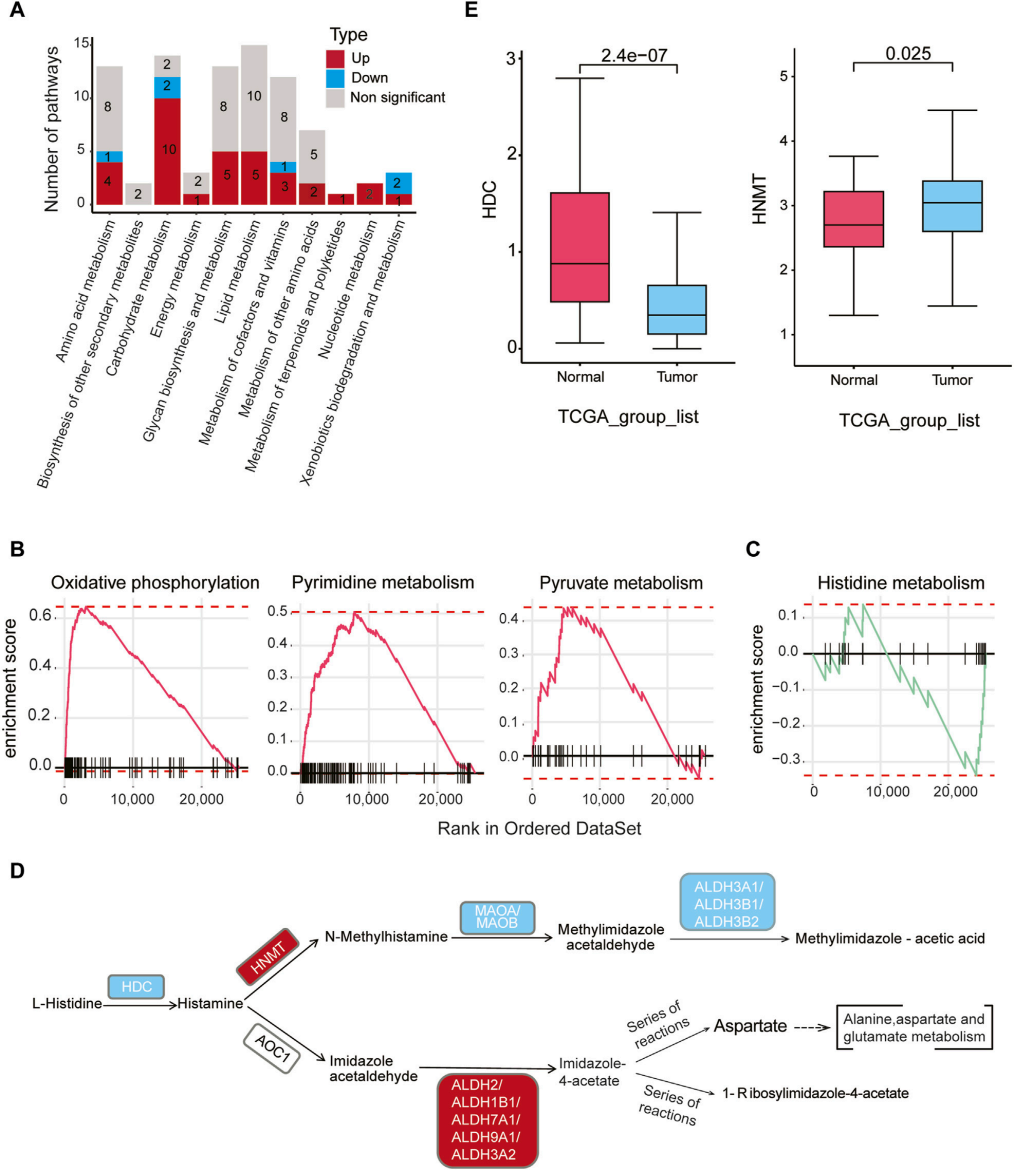
Metabolic dysregulation in malignant cells in gastric cancer **(A)** The number of significantly upregulated or downregulated metabolic pathways (*padj < 0.05*) between malignant cells in tumor samples and epithelial cells in normal samples within each of the 11 metabolic categories. Red indicates upregulation, blue indicates downregulation, and gray represents non-significant changes. **(B)** GSEA analysis reveals a significant upregulation of oxidative phosphorylation, pyrimidine metabolism, and pyruvate metabolism in malignant cells of tumor samples compared to normal epithelial cells. **(C)** GSEA analysis reveals a significant downregulation of the histidine metabolism pathway in malignant cells compared to normal epithelial cells. **(D)** Differential genes (gastric cancer malignant cells vs. normal epithelial cells) were mapped to the KEGG histidine metabolism pathway. Red indicates upregulation of genes in malignant cells, while blue indicates downregulation in malignant cells (*padj* < 0.05, |logFC| > 0.1). **(E)** According to bulk RNA-seq data from TCGA gastric cancer, *HDC* gene expression levels are higher in normal samples, while *HNMT* gene expression levels are higher in tumor samples.

We conducted a comprehensive analysis of the metabolic activities in malignant epithelial cells relative to normal epithelial cells, utilizing Gene Set Enrichment Analysis (GSEA). Our findings revealed 34 upregulated and six downregulated metabolic pathways within 11 distinct metabolic categories (*padj < 0.05*) (Figure 3A, Supplementary Table S7). Noteworthy, upregulated pathways in malignant epithelial cells included oxidative phosphorylation, pyrimidine metabolism, and pyruvate metabolism (Figure 3B). Conversely, downregulated pathways encompassed metabolism of xenobiotics by cytochrome P450, retinol metabolism, histidine metabolism, pentose and glucuronate interconversions, and ascorbate and aldarate metabolism (Supplementary Figure S5A).

Of particular interest, the histidine metabolism pathway exhibited downregulation in gastric cancer (Figure 3C). Histamine, a key regulator of tissue proliferation, is synthesized by histidine decarboxylase (*HDC*). Bioinformatics analysis revealed that the expression level of HDC was downregulated in cancer epithelial cells compared to normal cells (logFC = −1.25, *padj* < 0.05). Notably, *HNMT* gene expression was significantly upregulated in malignant epithelial cells, while downstream genes such as *MAOA, MAOB, ALDH3A1, ALDH3B1*, and *ALDH3B2* were downregulated (Figure 3D, *padj* < 0.05, Supplementary Table S8). Verification using the bulk RNA-seq data from TCGA confirmed lower *HDC* and higher *HNMT* expression in tumor samples compared to normal samples (Figure 3E), consistent with the scRNA-seq findings. Collectively, these results suggest a reduced histamine level in malignant epithelial cells compared to normal epithelial cells.

### High metabolic subcluster of malignant cells associated with better prognosis

**FIGURE 4:**
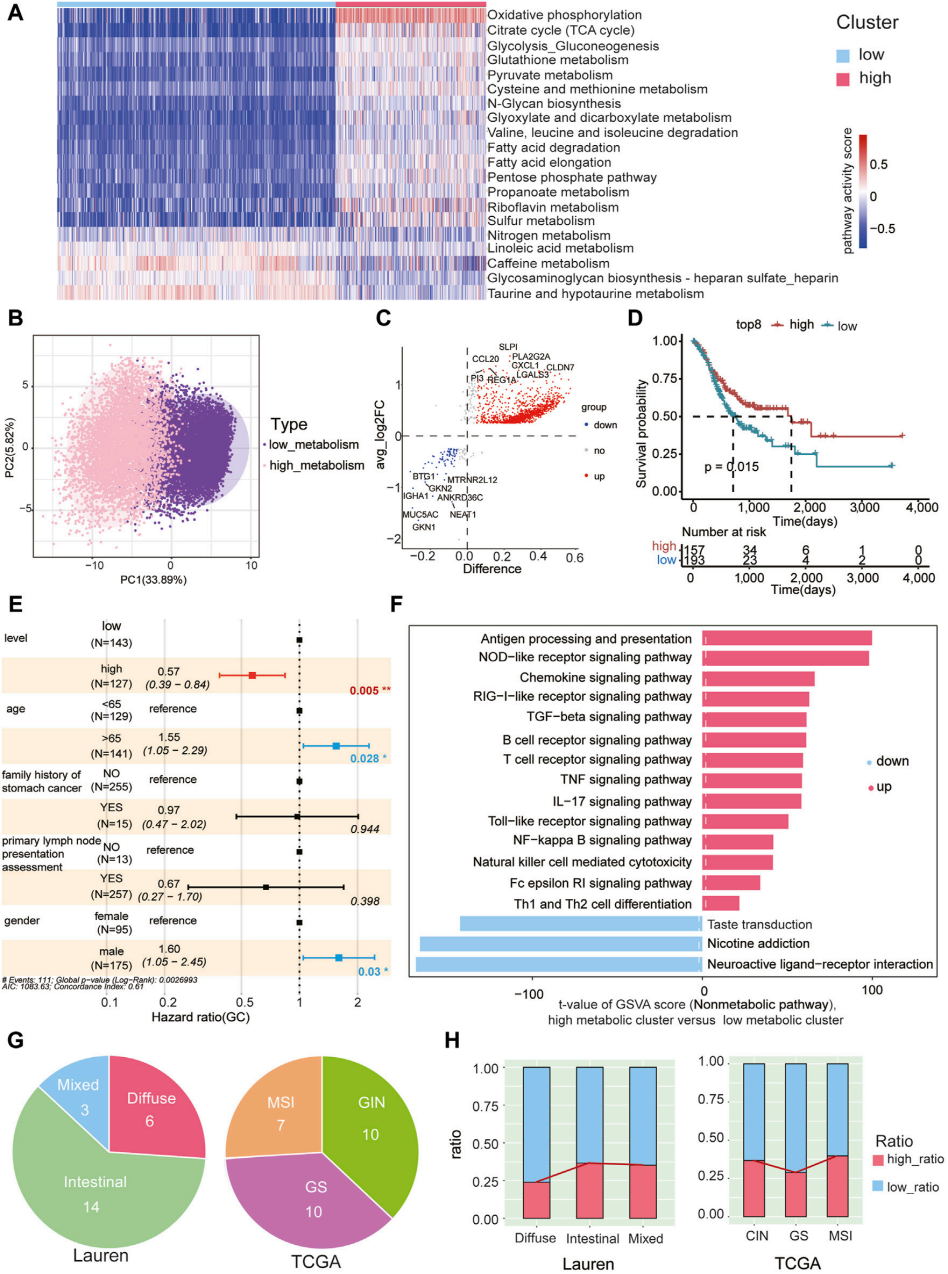
High metabolic subcluster of malignant cells associated with better prognosis **(A)** Unsupervised clustering of malignant cells in gastric cancer tumor samples based on GSVA metabolic pathway scores, dividing them into high metabolic (in red) and low metabolic subclusters (in blue). Each column in the heatmap represents malignant cells in each tumor sample, and each row represents the GSVA score of each pathway. **(B)** Principal component analysis was conducted on the GSVA scores of 85 metabolic pathways in each malignant cell, revealing significant metabolic pattern differences between high metabolic subclusters and low metabolic subclusters. **(C)** Characterization of differentially expressed genes between high metabolic and low metabolic subclusters. Red dots represent genes with high expression in the high metabolic subcluster, blue dots represent genes with high expression in the low metabolic subcluster, and gray dots represent genes with no significant difference. The top 8 DEG genes for both high and low metabolic subclusters are labeled. **(D)** The TCGA gastric cancer cohort was stratified into high and low groups based on the GSVA scores of the gene set using the surv_cutpoint function from the survminer package. Survival plots indicate a significant improvement in prognosis associated with the high-expression gene group. **(E)** The GSVA enrichment score of the high metabolic subcluster is an independent prognostic factor for overall survival (OS) in gastric cancer patients (*p* = 0.05). **(F)** The ranking of GSVA scores of non-metabolic pathways within high metabolic and low metabolic subclusters. Red indicates upregulated pathways and blue indicates downregulated pathways in the high metabolic subcluster, respectively. The *x*-axis represents the t-values obtained from the analysis of differential pathways between the high metabolic and low metabolic clusters of tumor cells. The magnitude of the t-value reflects the extent of difference in each pathway between the high metabolic and low metabolic clusters, with larger values indicating more significant differences. **(G)** The Lauren classification (left) and TCGA classification (right) of patients used in the study. **(H)** The proportion of cells belonging to the high and low metabolic subclusters in each Lauren subtype (left) and TCGA subtype (right).

Metabolic pathway scores were assigned to epithelial cells in tumor samples, leading to the formation of two clusters through unsupervised clustering based on 85 KEGG metabolic pathways (Supplementary Table S9). These clusters were denoted as low metabolic subcluster and high metabolic subcluster. Notably, oxidative phosphorylation exhibited significantly higher scores in the high metabolic subcluster, while taurine and hypotaurine metabolism scores were lower (Figure 4A). By performing PCA analysis on the GSVA scores of 85 metabolic pathways in each malignant cell, we observed significant metabolic pattern differences between high metabolic subclusters and low metabolic subclusters. This further confirms the reliability of our classification of high and low metabolic subclusters (Figure 4B).

Differential gene expression analysis between the high and low metabolic subclusters identified 1,367 upregulated and 104 downregulated genes (Figure 4C, Supplementary Table S10). Using the top eight upregulated genes (*SLPI, PLA2G2A, CXCL1, CCL20, REG1A, CLDN7, PI3, LGALS3*) as a representative gene set for the high metabolic subcluster, we calculated the GSVA score of this gene set for each patient in the TCGA gastric cancer cohort. Classification based on these scores in the TCGA cohort unveiled a significant association, indicating that patients with higher scores were correlated with a markedly improved prognosis (Figure 4D). Multivariate Cox proportional hazard analysis also demonstrated that the GSVA enrichment score of the representative gene set from the high metabolic subcluster independently predicted improved overall survival (OS) in gastric cancer. This significance persisted after adjusting for variables including age, family history of stomach cancer, primary lymph node presentation assessment, and gender (*p* = 0.005, Figure 4E). Additionally, non-metabolic KEGG pathways were also scored using GSVA in both high and low metabolic subclusters (Supplementary Table S11). Notably, immune pathways exhibited higher scores in the high metabolic subcluster, including antigen processing and presentation, NOD−like receptor signaling pathway, chemokine signaling pathway (Figure 4F). These findings suggest that epithelial cells with higher metabolic scores have the potential to induce a stronger immune response, correlating with an improved prognosis.

To further explore the correlation between the proportions of high metabolic subclusters and immune cell subpopulations, we depicted the expression trends of high metabolic subclusters and five other immune cell types using a line chart (Supplementary Figure S6A). However, we did not observe a significant correlation between metabolic levels and the proportions of immune cells (Supplementary Figure S6B).

We further conducted a detailed examination of the correlation between the prevalence of high metabolic subclusters in patients categorized under the classical clinical Lauren or TCGA classification (Figure 4G). We observed that the proportion of high metabolic subclusters is lower in diffuse-type tumors and higher in intestinal-type tumors (chi-square test, *padj* < 0.05, except for Intestinal Versus Mixed) (Figure 4H, Supplementary Table S12), aligning with the better prognosis for patients with the intestinal type (Qiu et al., 2013). Previous studies indicated that the prognosis for patients with MSI, CIN, and GS subtypes follows the order of MSI > CIN > GS (Sohn et al., 2017). Similarly, our findings revealed that the proportion of high metabolic subcluster in the GS subtype is lower than that in CIN and MSI subtypes (Figure 4H). Statistical analysis of chi-square test indicated the differences in the proportions of high metabolic subcluster among TCGA subtypes are significant (*padj* < 0.05, Supplementary Table S13).

### Identification of CSC-like cells associated with poor prognosis within the low metabolic subcluster

**FIGURE 5:**
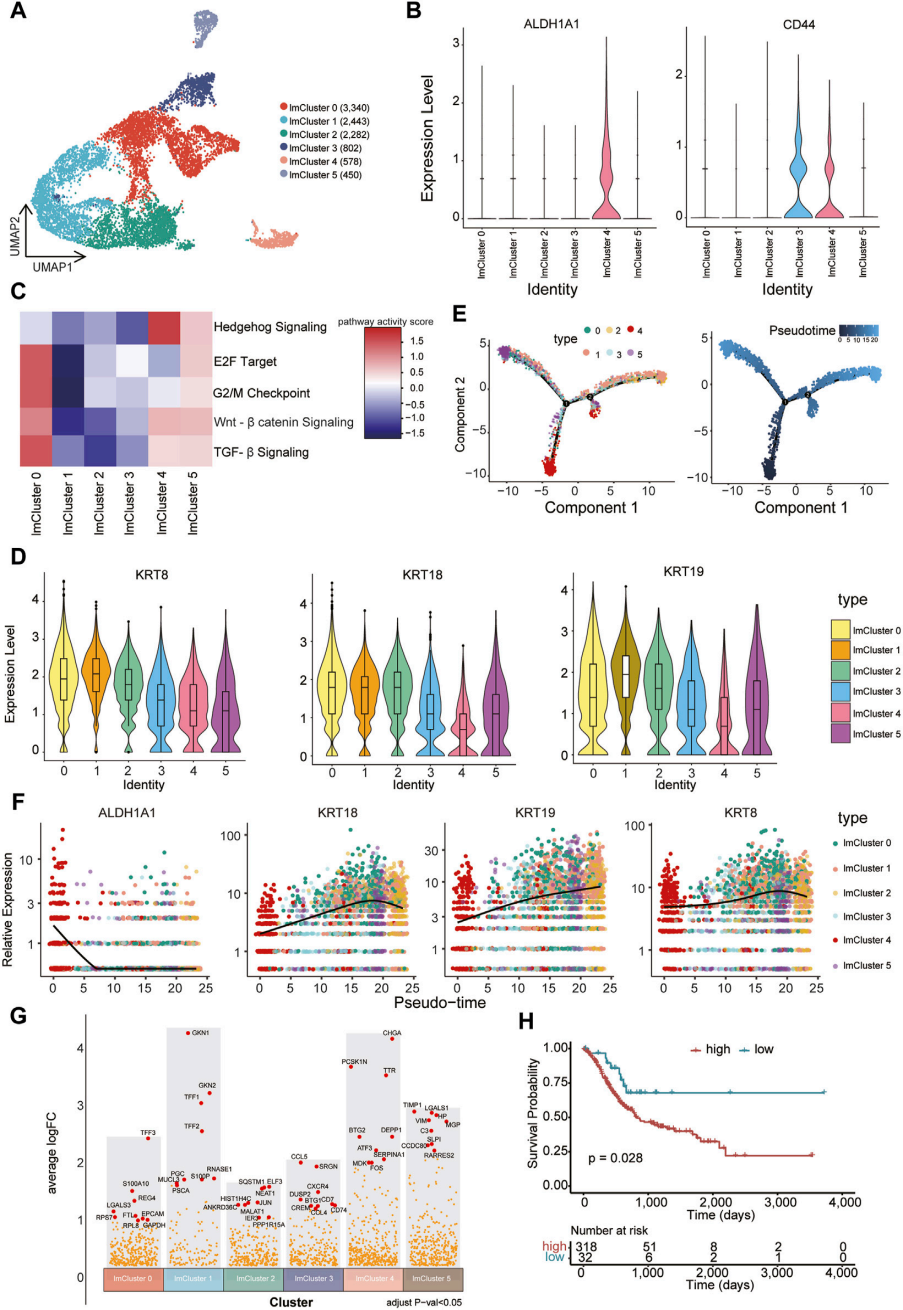
Identification of CSC-like cells associated with poor prognosis **(A)** Cells belonging to the low metabolic subcluster were reclustered into six subsets, namely, lmCluster0-5. **(B)** The expression levels of representative stem cell markers (*ALDH1A1* and *CD44*) in the lmClusters are shown. **(C)** The GSEA scores of the representative stem cell-related signaling pathways in the lmClusters are shown in the heatmap. **(D)** The violin plots display the expression levels of *KRT8, KRT18*, and *KRT19* in the lmClusters, while the boxplots depict the median, quartiles, and range of the genes. **(E)** The pseudotime trajectories for the lmClusters are shown, each dot represents 1 cell. **(F)** The expression levels of *ALDH1A1, KRT8, KRT18,* and *KRT19* genes are shown in the lmClusters at the indicated pseudotime. **(G)** The differentially expressed genes were identified in the lmClusters. The top nine highly expressed genes in each subset are denoted in red dots (*padj* < 0.05), while other differentially expressed genes are represented by orange dots. **(H)** The top nine differentially expressed genes (DEGs) in lmCluster4 are selected as a gene set representing this subcluster. Classification of the TCGA gastric cancer cohort into high and low groups is based on the GSVA scores of the gene set using the surv_cutpoint function from the survminer package. The survival plot indicates that the group with higher expression of the gene set is associated with a poorer prognosis.

CSCs represent a distinct, self-renewing, and tumorigenic subpopulation within tumors, playing a pivotal role in tumor persistence, relapse, and metastasis (Pattabiraman and Weinberg, 2014). Compared to the normal cancer cell, CSCs generally display a quiescent or slow-cycling state. A number of cell surface markers such as *CD44* and aldehyde dehydrogenase (*ALDH1A1*) are often used to identify and enrich CSCs (Batlle and Clevers, 2017). We further subjected cells within the low metabolic subcluster to a refined analysis, resulting in the delineation of six distinct groups (lmCluster0-5) (Figure 5A). Remarkably, the expression of CSCs marker genes was notably elevated in lmCluster4 (Figure 5B). GSEA analysis revealed heightened activity in stem cell-related signaling pathways in lmCluster4, including the transforming growth factor (TGF)-β pathway, hedgehog pathway, and Wnt-β pathway, within lmCluster4 (Pattabiraman and Weinberg, 2014) (Figure 5C). Additionally, the levels of pathways related to the cell cycle, such as E2F target and G2/M checkpoint, are notably diminished in lmCluster4 (Figure 5C). This observation aligns with the characteristic quiescent or slow-cycling state typically associated with stem cells. We also revealed lower expression levels of *KRT8, KRT18,* and *KRT19* in lmCluster4 (Figure 5D). Notably, studies have reported a significant correlation between low expression of *KRT19* and poor prognosis in breast cancer (Saha et al., 2018). Temporal analysis revealed that lmCluster4 marks the initiation of the cell differentiation trajectory (Figure 5E). Of note, throughout the development stages, lmCluster4 exhibits the highest expression of the CSCs marker gene *ALDH1A1*, while concurrently displaying the lowest levels of *KRT8, KRT18*, and *KRT19* genes (Figure 5F). Using the top nine upregulated genes (*CHGA, PCSK1N, TTR, DEPP1, BTG2, ATF3, SERPINA1, MDK, FOS*) as a representative gene set for the lmCluster4 (Figure 5G), we calculated the GSVA score of this gene set for each patient in the TCGA gastric cancer cohort. Classification based on these scores in the TCGA cohort revealed that patients with higher scores were associated with a worse prognosis (Figure 5H). These results suggest lmCluster4 represents stem cell-like cells and is associated with a worse prognosis.

## Discussion

Metabolic dysregulation has been linked to clinical outcomes and treatment responses in various cancers, recognized as a crucial driving factor in cancer development (Daemen et al., 2018; Gentric et al., 2019). Despite the non-direct correlation between metabolic gene expression and metabolic flux or metabolite abundance, evidence suggests that gene expression provides valuable insights into predicting metabolic flux and metabolite concentrations (Mehrmohamadi et al., 2014; Peng et al., 2018). This study aimed to characterize the metabolic features of gastric cancer at single-cell resolution, focusing on 85 metabolic pathways from the KEGG database, encompassing 1,667 metabolic genes. Utilizing high-quality scRNA-seq data (Kumar et al., 2022), we explored the metabolic characteristics of various cell subpopulations. The study identified distinct metabolic activities in both normal and tumor samples, with epithelial and chief cells exhibiting significantly higher metabolic activities (Figure 2B). Noteworthy, metabolic pathways displayed differential activities, potentially influenced by the microenvironment and physiological state of each cell type. Fibroblast cells in tumor samples, crucial for tumor microenvironment processes, exhibited higher metabolic activity than in normal samples (Kalluri, 2016). Detailed metabolic profiles indicated significantly higher pathway activities in tumor epithelial cells, with the histidine metabolism pathway showing downregulation. Histamine exhibits bidirectional effects in tumor progression, enhancing chronic inflammation, promoting immune evasion, and inducing stromal remodeling (Scolnick et al., 1984; Garcia-Caballero et al., 1989; Moya-García et al., 2021). However, some studies propose its potential to suppress reactive oxygen species, supporting immune regulation and enhancing tumor cell destruction (Hansson et al., 1996). The precise role of histamine in gastric cancer remains uncertain, emphasizing the need for further research to elucidate its impact on tumor development.

The metabolic profile of malignant epithelial cells significantly diverges from that of normal epithelial cells, with these metabolic abnormalities playing pivotal roles in tumor initiation, progression, and treatment resistance. A thorough examination of the metabolic characteristics of gastric cancer epithelial cells provides insights into how these cells adapt to the tumor microenvironment, secure energy, and proliferate. Employing GSVA to score each cell and subsequent unsupervised clustering facilitated the categorization of malignant cells into high-metabolic and low-metabolic subclusters. Notably, oxidative phosphorylation emerged as the most significantly different pathway between these subclusters (Figures 4A,B). Utilizing diverse analytical methods, we explored the relationship between the high-metabolic subcluster and patient prognosis. Survival analysis disclosed improved overall survival among patients in the high-metabolic subcluster. Multivariate Cox proportional hazards regression analysis confirmed that the GSVA enrichment score of the high metabolic subcluster is an independent prognostic factor. Moreover, this high metabolic subcluster demonstrated the potential to induce a robust immune response, correlating with a relatively favorable prognosis (Figure 4C). This discovery unveils new perspectives for further exploration of tumor treatment strategies and disease management.

In a previous CPTAC study, it was found that the enrichment of metabolic pathways correlates with immune-cold phenotypes. The study classified the entire tumor samples into immune-cold, cool, -warm, and -hot categories and then evaluated the enrichment of metabolic pathways (Geffen et al., 2023). Additionally, in the study “A novel metabolic subtype with S100A7 high expression represents poor prognosis and immuno-suppressive tumor microenvironment in bladder cancer”) (Cai Y. et al., 2023), bladder cancer patients were divided into two heterogeneous metabolic subtypes (MRSs): MRS1, characterized by inactive metabolic features but with an immune-infiltrated microenvironment; MRS2, characterized by upregulated lipid metabolism. It is evident that cellular metabolic activity has different conclusions in tumor immune research. Besides these studies, a series of research, such as “Bulk and single-cell transcriptome profiling reveal the metabolic heterogeneity in gastric cancer” (Tao et al., 2023) and “Bulk and single-cell transcriptome profiling reveal extracellular matrix mechanical regulation of lipid metabolism reprogramming through YAP/TEAD4/ACADL axis in hepatocellular carcinoma” (Cai J. et al., 2023), utilized bulk RNA data to group tumors, then subdivided them at the single-cell level based on the average expression values of all genes in individual cells from each patient. This approach may result in metabolic genes still being the average of many cell types. In contrast, in our study, clustering was directly performed using the metabolic gene set of malignant cells. This difference in methodology may lead to different interpretations regarding the enrichment of metabolic pathways and their association with immune phenomena. Direct clustering using the metabolic gene set of malignant cells may more directly reflect the metabolic characteristics of tumor cells. This approach highlights the importance of uncovering the association between tumor metabolic pathway enrichment and immune phenomena and its potential impact on developing new therapeutic strategies targeting tumor metabolic features. However, a more in-depth investigation is needed to unravel the subtle differences in these mechanisms and understand the complex relationship between the high-metabolic subcluster and the immune response.

CSCs represent a distinct, self-renewing, and tumorigenic subpopulation within tumors, playing a pivotal role in tumor persistence, relapse, and metastasis. The conventional assessment of cancer treatment efficacy, focusing on the ablation fraction of the tumor mass, may not fully address the distinct nature of CSCs due to their limited representation within the tumor bulk. Therefore, development of specific therapies targeted at CSCs holds hope for improvement of survival and quality of life of cancer patients (Pattabiraman and Weinberg, 2014). In the low metabolic subcluster, a subset of cells displaying CSCs-like characteristics was associated with a relatively poor prognosis. The identification of a stem cell-like population, particularly in the low metabolic subcluster (lmCluster4), highlights its significance in tumor biology. Elevated expression of CSCs marker genes, heightened activity in stem cell-related signaling pathways, and the initiation of the cell differentiation trajectory characterize lmCluster4. Importantly, the association of lmCluster4 with a worse prognosis emphasizes the potential clinical relevance of these stem cell-like cells (Figure 5H). Targeting such populations may be crucial for developing effective therapeutic strategies aimed at improving cancer patient outcomes.

## Conclusion

This study explored the metabolic landscape of gastric cancer at the single-cell level, examining 85 KEGG metabolic pathways. Utilizing high-quality scRNA-seq data, distinct metabolic activities were identified in various cell types. In-depth analysis categorized malignant cells into high- and low-metabolic subclusters, revealing oxidative phosphorylation as a significant differentiator. The high-metabolism subcluster demonstrated a correlation with improved overall survival and an enhanced immune response. Furthermore, our study identified a subset of cells within the low metabolic subcluster exhibiting CSCs-like characteristics, associated with a poorer prognosis. This comprehensive analysis enriches our understanding of the metabolic intricacies in gastric cancer, providing valuable insights for potential treatment strategies and disease management.

## Data Availability

Publicly available datasets were analyzed in this study. This data can be found here: Single-cell sequencing data for gastric cancer were obtained from the Gene Expression Omnibus (GEO), accession number GSE183904; Bulk RNA-seq data for gastric cancer (FPKM), along with patient clinical data, was sourced from the UCSC Xena database website (https://xenabrowser.net/datapages/).

## Supplementary material

SUPPLEMENTARY FIGURE S1 Non-epithelial cells in gastric cancer displayed in UMAP graphs with lineage-specific gene markers. **(A)** T cells marked by the expression of CD3D, CD3E, and CD2 expression. **(B)** NK cells marked by NKG7 and KLRD1 expression. **(C)** Myeloid cells identified by IL1B, C1QC, C1QB, C1QA, and FCER1G expression. **(D)** Plasma cells marked by IGLL5, JSRP1, TNFRSF17, and IGKC expression. **(E)** Mast cells marked by TPSAB1, TPSB2, KIT, and GATA2 expression. **(F)** B cells marked by MS4A1 expression. **(G)** Fibroblasts marked by DCN, COL1A2, COL1A1, LUM, and COL3A1 expression. **(H)** Endothelial cells marked by VWF, RAMP2, PECAM1, and PLVAP expression.

SUPPLEMENTARY FIGURE S2 Epithelial cells in gastric cancer displayed in UMAP graphs with cluster-specific gene markers. **(A)** Epithelial cells are marked by the expression of EPCAM, KRT18, KRT19, MUC1, MUC5AC, and KRT8. **(B)** Epithelial cells can be mainly divided into two clusters: cluster 1 and cluster 5. **(C)** Cluster 5 is defined as chief cells based on the expression of LIPF and PGC.

SUPPLEMENTARY FIGURE S3 Comparison of cell type proportions in gastric cancer and normal samples. **(A)** UMAP plot illustrating cell clusters in gastric cancer and normal samples. **(B)** Proportions of each cell type in tumor and normal samples, with each dot representing a sample.

SUPPLEMENTARY FIGURE S4 The metabolic landscape of cell types in normal samples. **(A)** Metabolic pathway activities in cell types from normal samples. Statistically non-significant values (random permutation test padj > 0.05) are left blank. **(B)** Distributions of pathway activities in different cell types from both gastric cancer and normal samples.

SUPPLEMENTARY FIGURE S5 Downregulated Pathways identified in malignant epithelial cells through Gene Set Enrichment Analysis (padj < 0.05).

SUPPLEMENTARY FIGURE S6 Correlation analysis between high metabolic subclusters and immune cells. **(A)** The line chart illustrates the expression trends of high metabolic subclusters and five other immune cell types. **(B)** The scatter plot depicts the correlation analysis between the proportion of high metabolic subclusters and B, Mast, Myeloid, Plasma, and TNK cells.

## Abbreviations

CSCs, Cancer stem cells; TCGA, The Cancer Genome Atlas; EBV, Epstein-Barr virus; MSI, Microsatellite instability; GS, Genomically stable; CIN, Chromosomal instability; TME, Tumor microenvironment; CTLs, Cytotoxic T Lymphocytes; scRNA-seq, Single-cell RNA sequencing; UMIs, Unique Molecular Identifiers; TPM, Transcripts per million; HVGs, Highly variable genes; FC, Fold change; tSNE, t-Distributed Stochastic Neighbor Embedding; PCA, Principal components analysis; UMAP, Uniform Manifold Approximation and Projection; DEGs, Differentially expressed genes; KEGG, Kyoto Encyclopedia of Genes and Genomes; GSEA, Gene Set Enrichment Analysis; GSVA, Gene Set Variation Analysis; HDC, Histidine decarboxylase; OS, Overall survival.
