# Supplementary figures and images for: Unraveling metabolic characteristics and clinical implications in gastric cancer through single-cell resolution analysis
Source: Front Mol Biosci. 2024 May 20;11:1399679. doi: 10.3389/fmolb.2024.1399679 (PMC11145399; doi:10.3389/fmolb.2024.1399679)

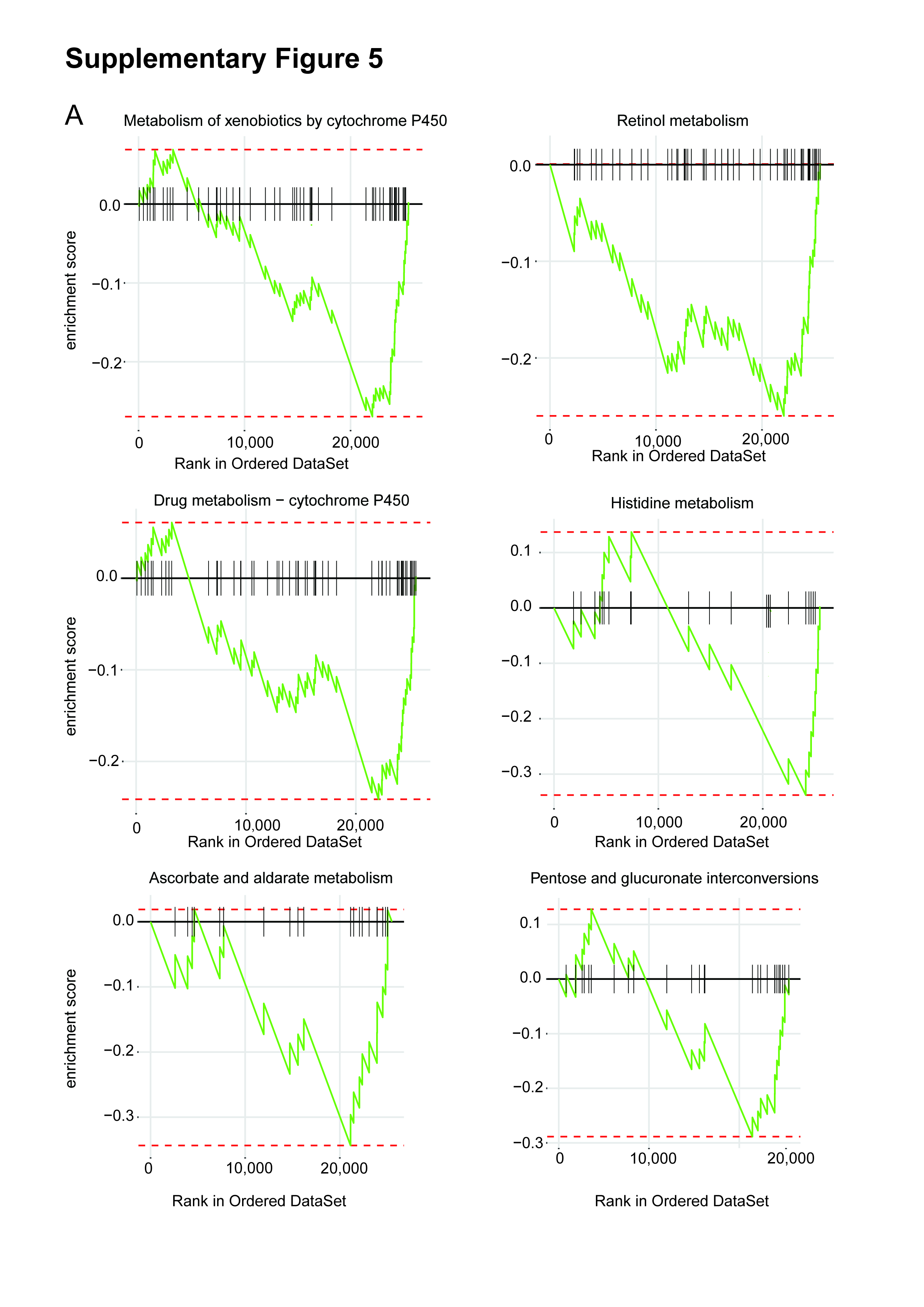

Supplement: Supplementary file 1 [file Image5.jpg]

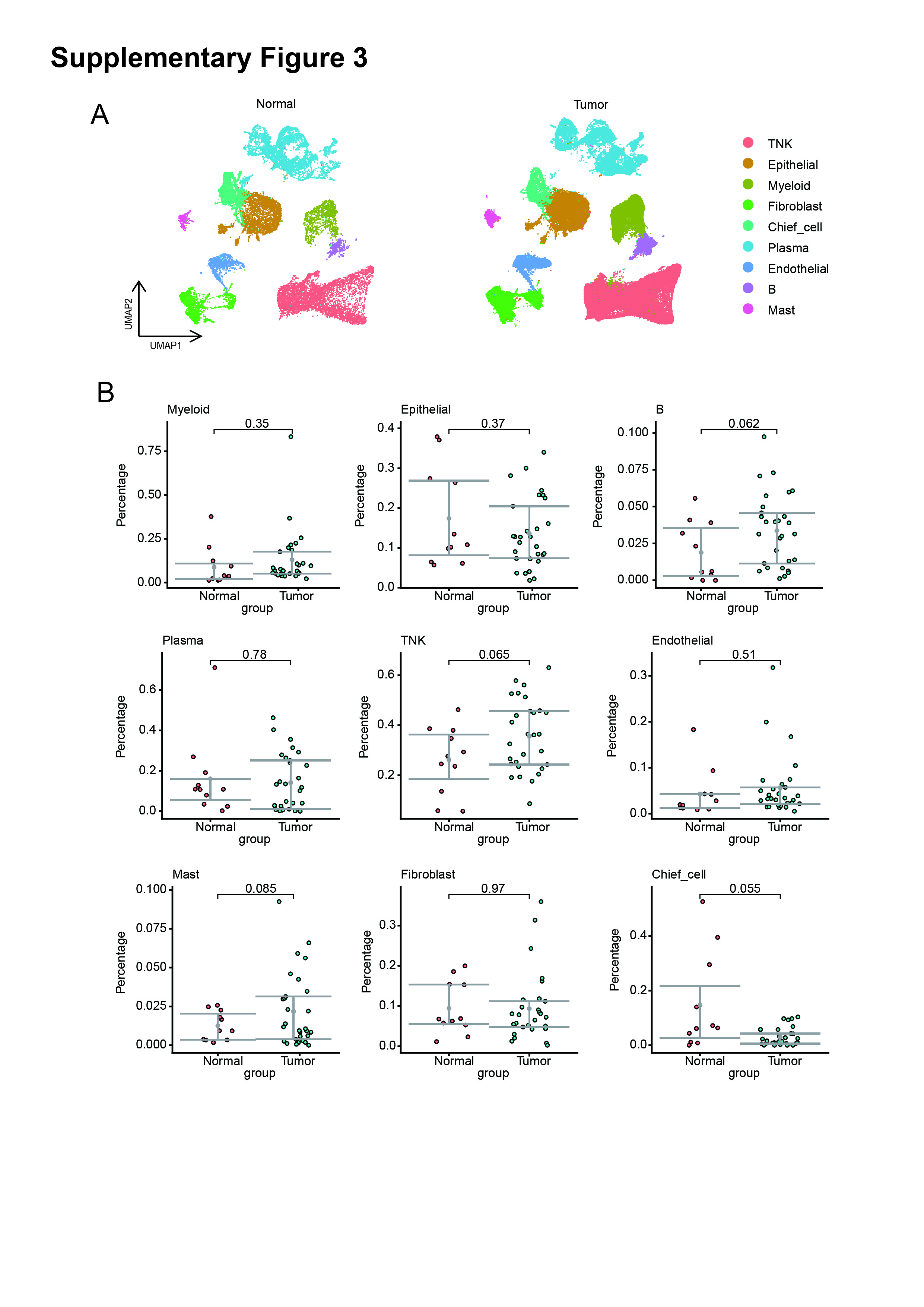

Supplement: Supplementary file 2 [file Image3.JPEG]

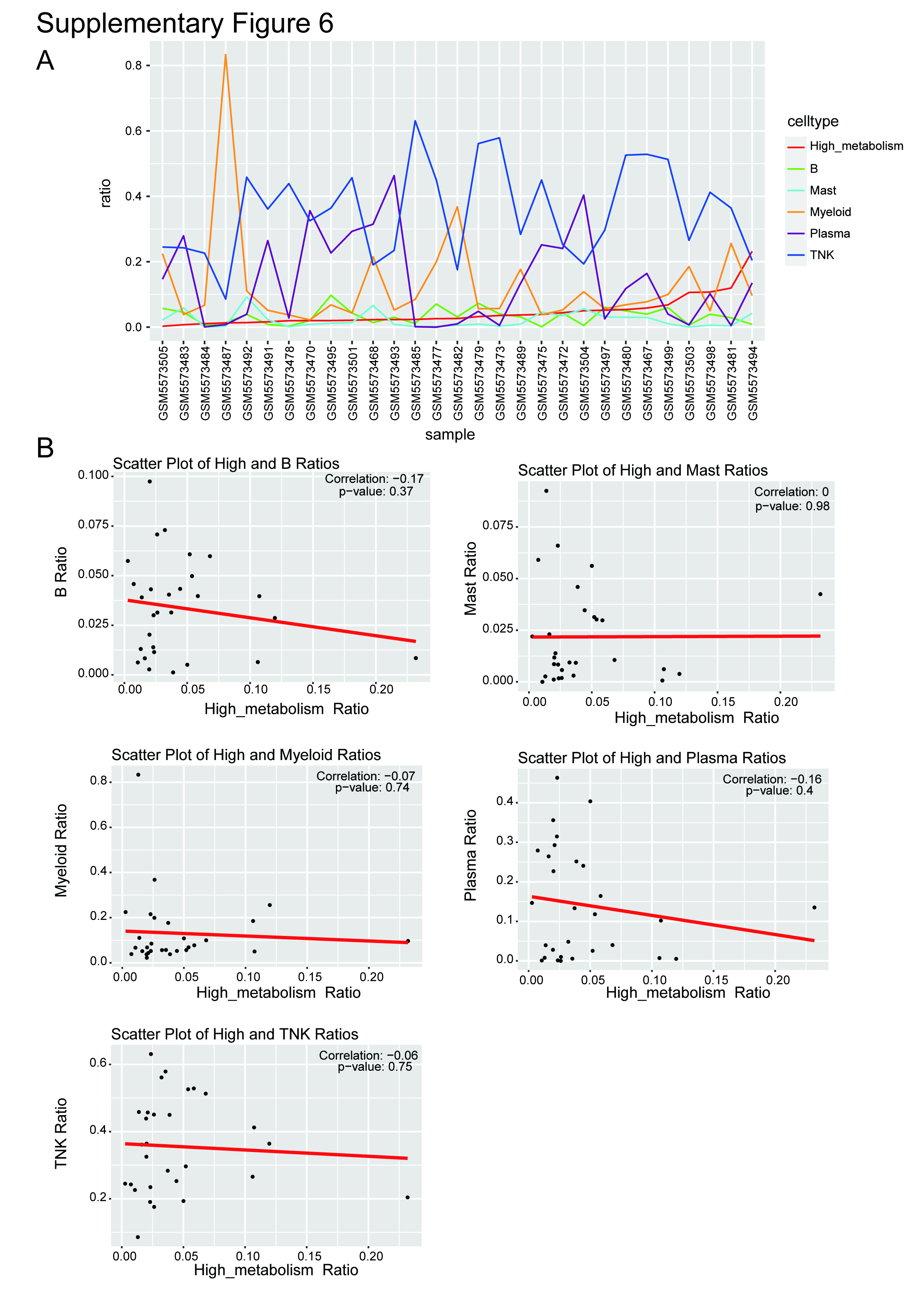

Supplement: Supplementary file 3 [file Image6.jpg]

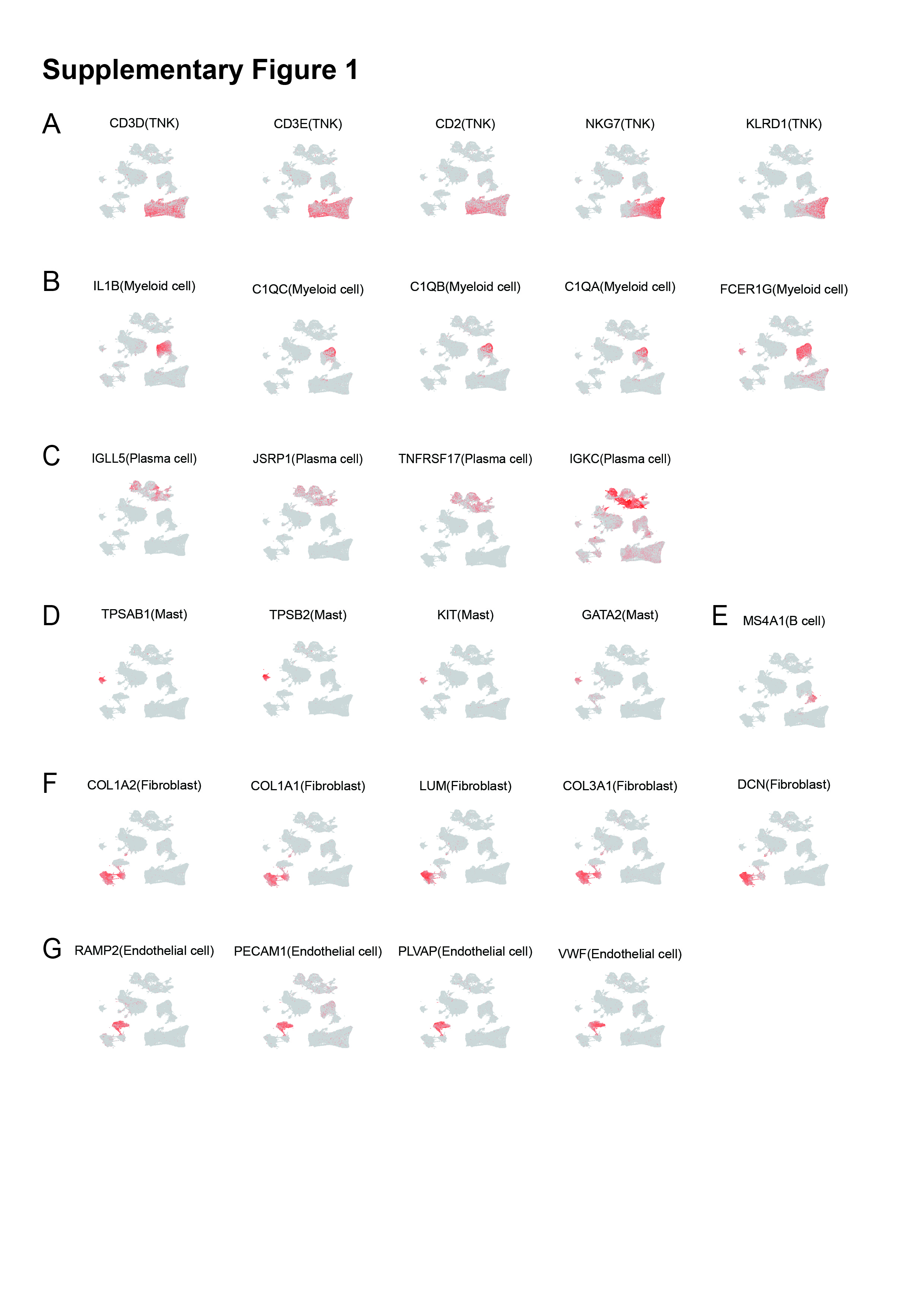

Supplement: Supplementary file 4 [file Image1.JPEG]

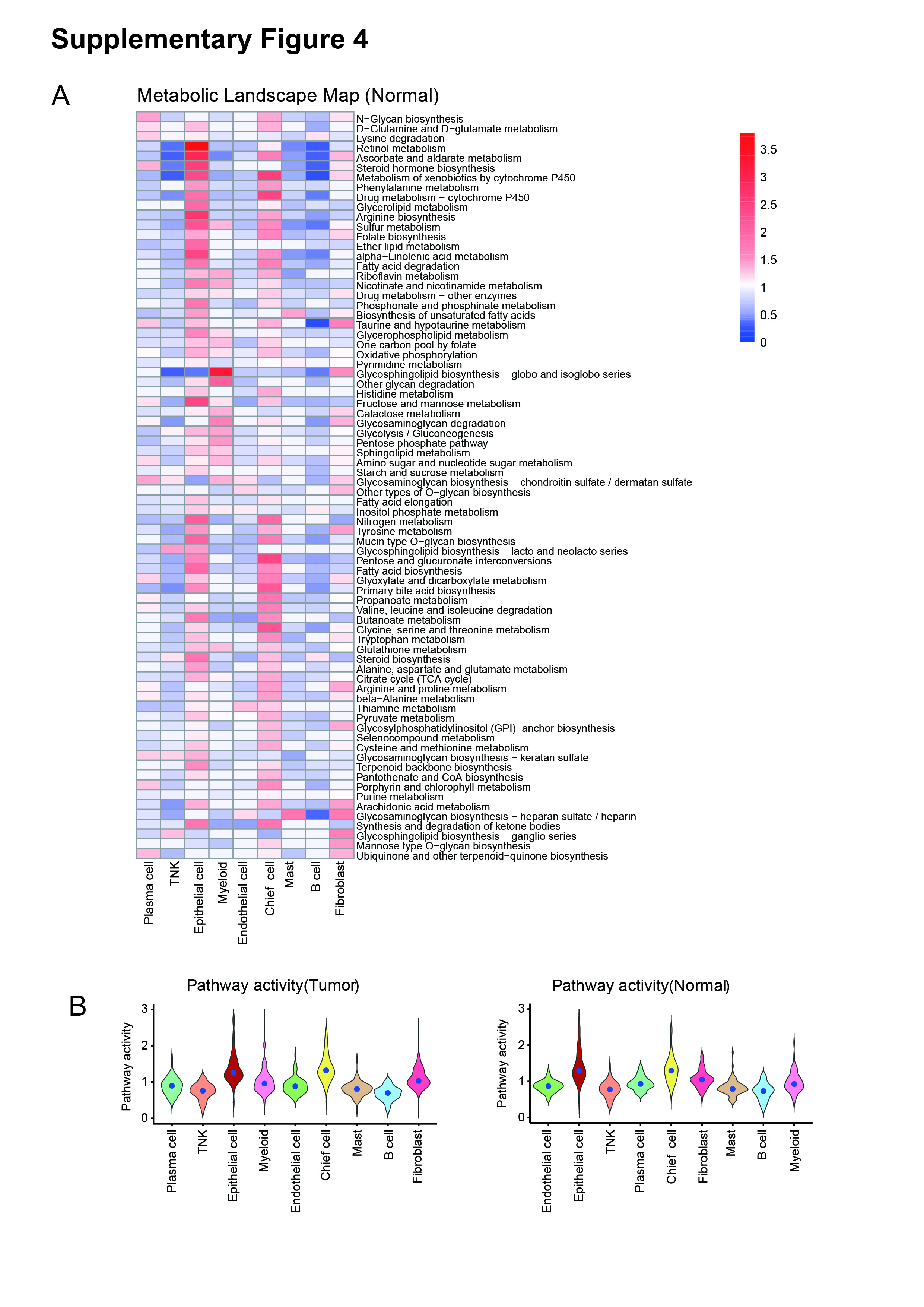

Supplement: Supplementary file 5 [file Image4.JPEG]

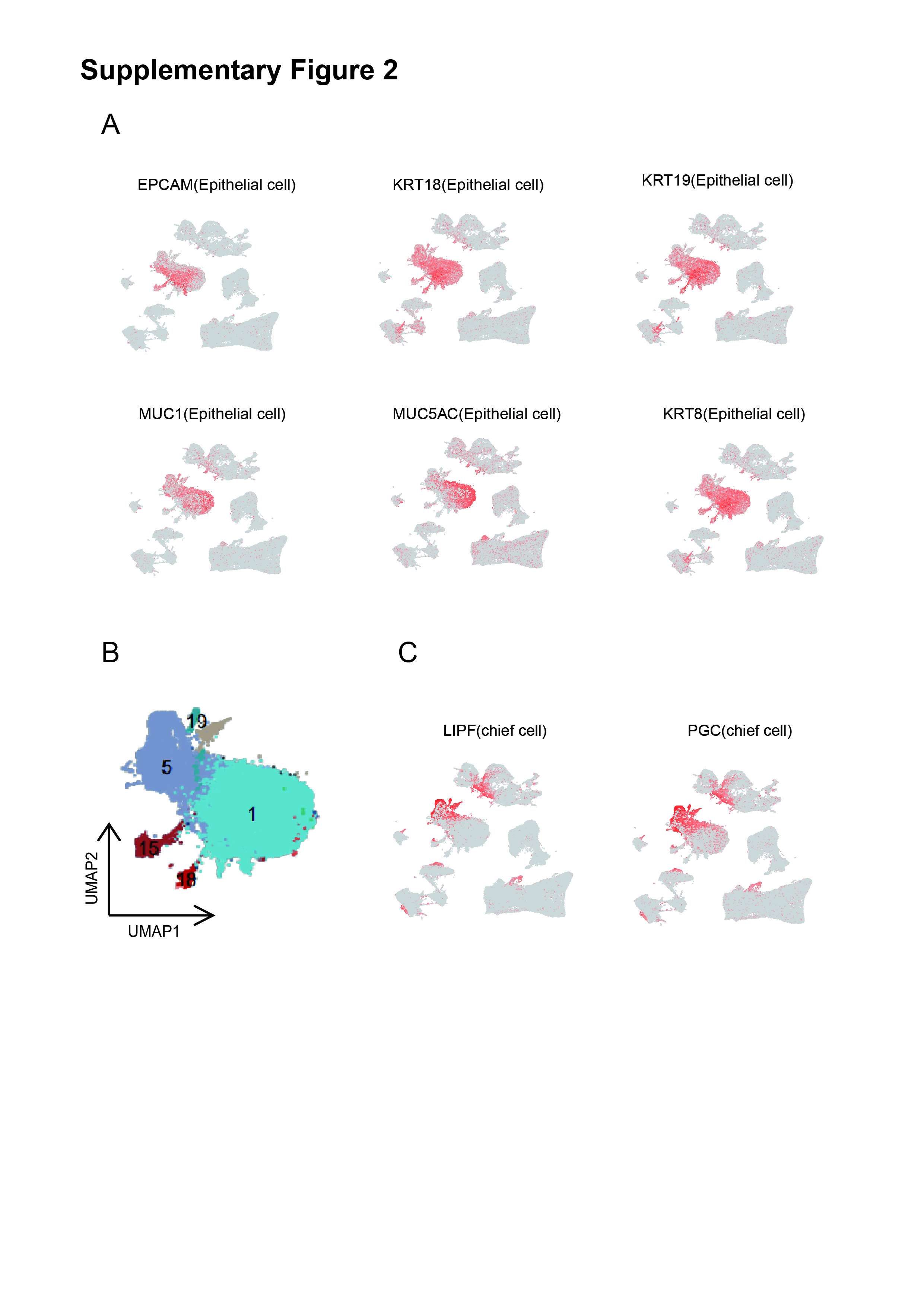

Supplement: Supplementary file 6 [file Image2.JPEG]
